# Systematic optimization of parameters involved in preparation of chromatin and chromatin immunoprecipitation (ChIP) workflow

**DOI:** 10.1186/1756-8935-6-S1-P122

**Published:** 2013-04-08

**Authors:** John M Rosenfeld, Tracy Cooke, Zirong Li, Kan Saito, Konstantin Taganov, Bhaskar Thyagarajan, Alejandra Solache

**Affiliations:** 1 Epigenetics R&D, EMD Millipore, Temecula, CA, USA

## 

The ChIP protocol is a fundamental tool for discoveries in the area of epigenomics and gaining insight into epigenetic phenomenon. Despite the fact that many vendors of life science research tools offer products to support ChIP, very little innovation has occurred around the ChIP method itself to make this process more reproducible and reliable. Issues surrounding antibody specificity and performance are critical to high resolution genomic mapping of chromatin events. In addition, reliable high-throughput options are not readily available for ChIP despite the ability to automate parts of the workflow. Chromatin preparation, for example, remains a laborious process and the field lacks accepted criteria to make determinations on the quality of chromatin suitable for ChIP.

We have engaged in an iterative process of examining parameters that influence chromatin fragmentation on a variety of devices and under various buffer systems, and have developed a protocol for optimizing this process for researchers new to the area of chromatin biology. As a leading antibody supplier, we have produced tools that allow researchers to evaluate the specificity of histone modifications antibodies in their own laboratories utilizing a simple dot blot western blotting approach. In addition, we have developed a new high-throughput and low-cell capable protocol that simplifies the process, reduces variability and significantly improves signal-to-noise ratio. This protocol is particularly suited for new users/beginners, and can utilize chromatin derived from cells and tissue. We present data comparing the effect of different sonication protocols on ChIP, analysis of user-to-user variation, as well as methods to optimize and control for factors that introduce variability in ChIP.

